# Comparison of the Cytotoxic Potential of Cigarette Smoke and Electronic Cigarette Vapour Extract on Cultured Myocardial Cells

**DOI:** 10.3390/ijerph10105146

**Published:** 2013-10-16

**Authors:** Konstantinos E. Farsalinos, Giorgio Romagna, Elena Allifranchini, Emiliano Ripamonti, Elena Bocchietto, Stefano Todeschi, Dimitris Tsiapras, Stamatis Kyrzopoulos, Vassilis Voudris

**Affiliations:** 1 Onassis Cardiac Surgery Center, Sygrou 356, Kallithea 17674, Greece; E-Mails: dtsiapras@hotmail.com (D.T.); stkyrz@gmail.com (S.K.); vvoudris@otenet.gr (V.V.); 2 ABICH S.r.l, Biological and Chemical Toxicology Research Laboratory, Via 42 Martiri, 213/B-28924 Verbania (VB), Italy; E-Mails: giorgio.romagna@gmail.com (G.R.); Elena.Allifranchini@abich.it (E.A.); emiliano.ripamonti@abich.it (E.R.); elena.bocchietto@abich.it (E.B.); stefano.todeschi@abich.it (S.T.)

**Keywords:** electronic cigarette, smoking, tobacco, nicotine, cytotoxicity, myocardial cell, public health

## Abstract

*Background:* Electronic cigarettes (ECs) have been marketed as an alternative-to-smoking habit. Besides chemical studies of the content of EC liquids or vapour, little research has been conducted on their *in vitro* effects. Smoking is an important risk factor for cardiovascular disease and cigarette smoke (CS) has well-established cytotoxic effects on myocardial cells. The purpose of this study was to evaluate the cytotoxic potential of the vapour of 20 EC liquid samples and a “base” liquid sample (50% glycerol and 50% propylene glycol, with no nicotine or flavourings) on cultured myocardial cells. Included were 4 samples produced by using cured tobacco leaves in order to extract the tobacco flavour. *Methods:* Cytotoxicity was tested according to the ISO 10993-5 standard. By activating an EC device at 3.7 volts (6.2 watts—all samples, including the “base” liquid) and at 4.5 volts (9.2 watts—four randomly selected samples), 200 mg of liquid evaporated and was extracted in 20 mL of culture medium. Cigarette smoke (CS) extract from three tobacco cigarettes was produced according to ISO 3308 method (2 s puffs of 35 mL volume, one puff every 60 s). The extracts, undiluted (100%) and in four dilutions (50%, 25%, 12.5%, and 6.25%), were applied to myocardial cells (H9c2); percent-viability was measured after 24 h incubation. According to ISO 10993-5, viability of <70% was considered cytotoxic. *Results:* CS extract was cytotoxic at extract concentrations >6.25% (viability: 76.9 ± 2.0% at 6.25%, 38.2 ± 0.5% at 12.5%, 3.1 ± 0.2% at 25%, 5.2 ± 0.8% at 50%, and 3.9 ± 0.2% at 100% extract concentration). Three EC extracts (produced by tobacco leaves) were cytotoxic at 100% and 50% extract concentrations (viability range: 2.2%–39.1% and 7.4%–66.9% respectively) and one (“Cinnamon-Cookies” flavour) was cytotoxic at 100% concentration only (viability: 64.8 ± 2.5%). Inhibitory concentration 50 was >3 times lower in CS extract compared to the worst-performing EC vapour extract. For EC extracts produced by high-voltage and energy, viability was reduced but no sample was cytotoxic according to ISO 10993-5 definition. Vapour produced by the “base” liquid was not cytotoxic at any extract concentration. Cell survival was not associated with nicotine concentration of EC liquids. *Conclusions:* This study indicates that some EC samples have cytotoxic properties on cultured cardiomyoblasts, associated with the production process and materials used in flavourings. However, all EC vapour extracts were significantly less cytotoxic compared to CS extract.

## 1. Introduction

Smoking is a major risk factor for cardiovascular disease [1]. Although several pharmaceutical products are currently available for smoking cessation, long term quit-rates are relatively low [2,3]. Tobacco harm reduction is a strategy of reducing smoking-relating harm by using safer sources of nicotine for smokers unable or unwilling to quit [4]. Electronic cigarettes (ECs) have been introduced to the market in recent years as an alternative-to-smoking habit. They usually consist of a battery and an atomiser where liquid is stored and vaporises by activating the battery. They are the only products in tobacco harm reduction that do not contain tobacco, excluding nicotine replacement therapies which are currently approved for short term use and with the goal to treat nicotine addiction. Awareness and use of these products have increased significantly [5], and this has raised global debate and controversy [6]. Several organisations such as World Health Organisation and Food and Drug Administration have expressed concerns about the health effects of using ECs [7,8].

There is significant evidence of the cytotoxic potential of tobacco cigarettes. Oxidative stress is an important associated mechanism, with each puff of smoke containing 10^15^ molecules of free radicals [9]. This can have direct toxic effects on a variety of cells, including myocardial cells [10]. Several chemicals causing oxidative stress are produced during cigarette smoking by the combustion process. No combustion is involved in EC use; the liquid is heated by delivering electrical current to a resistance inside the atomiser, and the resulting vapour is subsequently inhaled by the user. In recent years, new generation devices have been developed, producing more vapour mainly by using higher-capacity batteries and by delivering higher energy (wattage) to the resistance. However, it is unknown whether free radicals are present in EC vapour, and the cytotoxic potential of EC liquids has not been adequately studied. Therefore, the purpose of this study was to evaluate the cytotoxic potential of EC vapour from a variety of liquid samples on cultured cardiomyoblasts, and to examine whether higher wattage has any effect in their cytotoxic potential. 

## 2. Experimental Section

### 2.1. Materials

**Figure 1:**
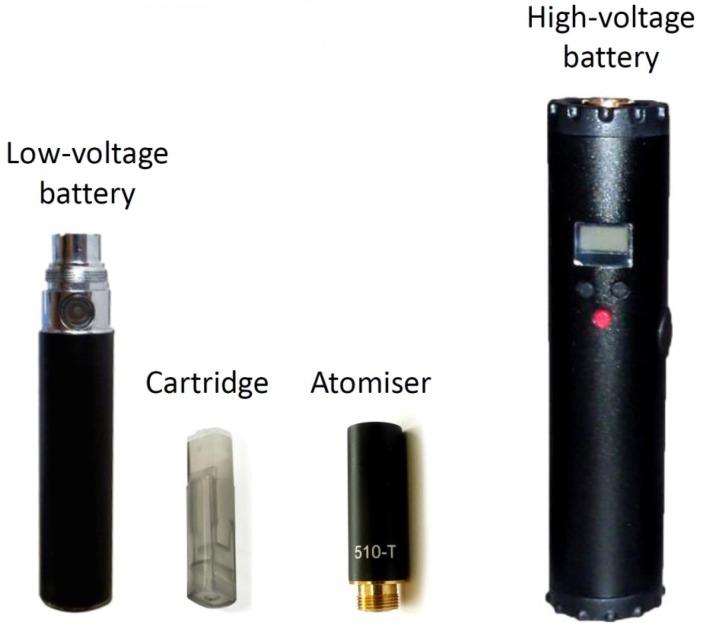
Electronic cigarette devices used in this study. The low voltage device delivered 3.7 volts to the atomiser while the high-voltage device integrates an electronic circuit by which the voltage applied to the atomiser can be adjusted. For the high-voltage experiments it was set to 4.7 volts. The cartridge is the part where liquid is stored, while the atomiser is the part where the resistance and wick are placed and evaporation of the liquid takes place.

A commercially available tobacco cigarette with 0.8 mg nicotine, 10 mg tar and 10 mg carbon monoxide yields was used for this experiment (Marlboro, Philip Morris Italia S.r.l., Rome, Italy). Twenty commercially-available liquids used for ECs were obtained from the market in sealed bottles, manufactured or distributed by five different companies. Nicotine concentrations varied from 6 mg/mL to 24 mg/mL (Table 1). Seventeen of them were tobacco flavours, and three were sweet or fruit flavours. The liquids were mainly composed of propylene glycol, glycerol, nicotine and a variety of substances usually approved for use in food flavouring industry. Most of the companies reported using tobacco absolute extract (also called natural tobacco extract) as additive in tobacco-flavoured liquids. This flavouring is not approved for use in food. One manufacturer (House of Liquid, Nottingham, UK) uses a different production method. Cured tobacco leaves are inserted into bottles containing propylene glycol and glycerol, and are allowed to rest for several days. Subsequently, the leaves are removed and the liquid is filtered and bottled for use with ECs. The same process but different tobacco blends are used for producing the four samples tested (according to manufacturer’s website: www.houseofliquid.com). Additionally, a “base” EC sample, consisting of 50% propylene glycol and 50% glycerol (without nicotine or any flavouring) was tested. In order to choose the samples for the analysis, an online poll in an EC users’ forum about the popularity of liquids from four major manufacturers/retailers in Greece was organized by the researchers; additionally, data on sales volume were requested from the retailers. The tobacco leaves-produced liquids were chosen because the procedure used in the production process is unique and such liquids are popular in the Greek market and abroad. Two sets of experiments were performed; one using regular voltage and a second using higher voltage for EC vapour production. For the first set, a commercially available EC device consisting of a lithium battery (eGo, Joyetech, Shenzhen, China), a 2.2-Ohms atomiser (510 T, Omega Vape, Manchester, UK) and a tank-type cartridge where liquid is stored were used (Figure 1). The battery was fully charged before each extract production and was measured to deliver 3.7 volts with the atomiser attached. A new atomiser was used for each vapour extract production. Before use, the atomiser was cleaned with ultrasound and distilled water, to remove the liquid substance that is used during the manufacturing process. Subsequently, its resistance was measured by a digital multimeter and it was discarded if it was found to differ by more than 0.1 Ohm from the nominal value. The total energy applied to the atomiser for the first set of experiments was 6.2 watts. For the second setting, a variable-voltage device was used (Lavatube, Shenzhen, China). It consists of an aluminium tube where a rechargeable lithium battery is inserted, and incorporates an electronic circuit by which the voltage can be manually adjusted by pressing buttons (Figure 1). The atomiser-type used was similar to the first setting. The device was adjusted so that 4.5 volts were delivered to the atomiser. Thus, the total energy applied for vapour production in the second experimental setting was 9.2 watts. Before initiating the experiments, four samples were randomly chosen to be additionally tested using the high-voltage device. For every sample, a brand new atomiser and cartridge were used, to avoid contamination between samples. The batteries were fully-charged before each extract preparation.

An important issue that needs to be clarified before proceeding with laboratory experiments is the determination of the “dry puff” phenomenon [11]. It occurs when insufficient liquid is supplied to the wick of the atomiser, leading to temperature elevation. This is detected by the user as an unpleasant burning taste which is avoided by reducing puff duration and increasing interpuff interval. Therefore, if this phenomenon is reproduced in the laboratory setting it does not represent EC use in realistic conditions. Since no laboratory method has been developed to detect it, one of the researchers (who is an experienced EC user) was assigned to test both devices in order to detect the dry puff phenomenon. 

**Table 1:** Electronic cigarette liquids tested in this study.

Samples	Nicotine concentration (mg/mL)	Main ingredients ^a^	Distributor/Manufacturer
City	6	30% VG/60% PG	Alter Ego/El Greco
Americano	9	30% VG/60% PG	Alter Ego/El Greco
Tribeca	12	VG/PG *	Alter Ego/Halo
Classic	18	30% VG/60% PG	Alter Ego/El Greco
Cinnamon & Cookies	6	50% VG/50% PG	Atmos Lab
RY69	6	50% VG/50% PG	Atmos Lab
Green apple	12	50% VG/50% PG	Atmos Lab
Bebeka	18	50% VG/50% PG	Atmos Lab
Base ^b^	0	50% VG/50% PG	Flavourart
MaxBlend	9	85% VG	Flavourart
RY4	9	85% VG	Flavourart
Virginia	18	85% VG	Flavourart
El Toro Cigarrillos (1) ^c^	12	VG/PG *	House Of Liquid
El Toro Cigarrillos (2) ^c^	12	VG/PG *	House Of Liquid
Silverberry	12	VG/PG *	House Of Liquid
El Toro Guevara^ c^	18	VG/PG *	House Of Liquid
El Toro Puros^ c^	24	VG/PG *	House Of Liquid
Golden Margy	6	20% VG/80% PG	Nobacco
Golden Virginia	8	90% VG/10% PG	Nobacco
American Tobacco	11	90% VG/10% PG	Nobacco
Tobacco Echo	18	20% VG/80% PG	Nobacco

Abbreviations: VG, vegetable glycerol; PG, propylene glycol; **^a^** Approximate concentrations, according to manufacturers’ reports; **^b^** Sample consisting of propylene glycol and glycerol, without nicotine or flavourings; **^c^** Electronic cigarette samples made by using tobacco leaves; * Exact percentages were not disclosed by the manufacturers.

Both EC devices were tested on 4 s puffs [12]. It was found that the dry-puff phenomenon was consistently reproduced with the high-voltage device at 4 and 3 s puff duration. Therefore 2.5 s puffs were used with the high-voltage device, while 4 s puffs were used with the regular-voltage device.

### 2.2. Cell Cultures

Cytotoxicity was measured by MTT assay on monolayer-cultured H9c2 cardiomyoblast cells **(**ATCC CRL-1446**),** according to the ISO 10993-5 standard [13]. The reason for choosing this cell line was based on the better culture stability and reproducibility compared to human cardiomyocytes. Additionally, they have similar characteristics to adult cardiomyocytes [14] and have been extensively used as an *in vitro* model to study smoking-induced pathology [15,16,17]. Cells were grown in Dulbecco’s modified Eagle’s medium (Sigma-Aldrich, St. Louis, MO, USA), supplemented with fetal bovine serum (Euroclone, Milano, Italy), penicillin-streptomycin 0.1 g/mL (Euroclone) and kanamycin 0.1 g/mL (Sigma-Aldrich, St. Louis, MO, USA). The doubling time of the cell line was 24 ± 5 h. All related extracts, solutions and media were prepared using sterile laboratory conditions, components and ingredients. Routine checks for bacterial contamination were applied, in terms of both bacterial cultures and microscopic observation of the cell cultures after incubation for the presence of morphological changes typical of bacterial contamination.

### 2.3. Production of Extracts

Vapour extract was produced by simulating EC use. The EC atomiser cartridge was filled with 400 mg of liquid. The vapour was bubbled through an impinger by activating a vacuum pump and the EC device, resulting in 200 mg of liquid consumed (evaporated) and extracted in 20 mL of cultured medium (Dulbecco’s basal medium plus 10% fetal bovine serum). The vacuum pump was set so that each activation would consume approximately 4–5 mg of liquid, representing real use [12]. Weighing of the EC cartridge was performed before and during the experiment by a precision scale (Mettler, model AB104-S, precision of 0.1 mg) to make sure that 200 mg of liquid were consumed, leading to a final extract concentration of 1% (as dictated by ISO 10993-5 standard). The resulting solution was denoted as 100% EC vapour extract. For the regular-voltage experiments, each inhalation simulation lasted 4 s, with 60 s between inhalations; for the high-voltage experiments, each puff lasted 2.5 s. CS extract was produced by using the ISO 3308 standard (2 s puffs of 35 mL, one puff every 60 s) [18]. Three cigarettes were consumed, since it was previously found that this is a comparative measure to 200 mg of liquid [12]. The resulting solution was denoted as 100% CS extract. Immediately after preparation, all EC vapour and CS extracts were used in cell cultures.

### 2.4. Treatment and Exposure

Cells were seeded in 96-wells plate with Dulbecco’s basal medium plus 10% fetal bovine serum and maintained in culture for 24 h (5% CO_2_, 37 °C, >90% humidity) in order to form a semi-confluent monolayer. In each well, 100 μL of a cell suspension of 1 × 10^5^ cells/mL was dispensed. A different plate was prepared for each extract testing. On the next day, each plate was examined under the microscope to ensure that cell attachment was even across the plate. Subsequently, the medium was aspirated and replaced by medium containing the CS and EC liquid extracts in one undiluted (100%) and 4 diluted samples (50%, 25%, 12.5% and 6.25%). For the EC extract, 100% EC extract equals to a vapour extract concentration of 1%. Three different wells were treated with each dilution and columns 2 and 11 were used to culture cells with normal medium (without extract, untreated cells); then, they were incubated for 24 h at 37 °C. Subsequently, cells were tested for viability by MTT assay. Untreated cells were used as controls.

### 2.5. MTT Assay

The assay was performed according to the method developed by Mossman [19]. After incubation, the culture medium was removed and replaced with 10 μL of 1 mg/mL MTT. The cells were then incubated for 2 h. MTT (3-[4,5-dimethylthiazol-2-yl]-2,5-diphenyltetrazolium bromide) is cleaved by the mitochondrial dehydrogenases of viable cells leading to the formation of purple crystals, representing formazan metabolism, which are insoluble in aqueous solutions. The solution was then removed and replaced with 200 µL/well of isopropanol to extract and solubilize the formazan. It was incubated for 30 min at room temperature under medium speed shaking. Then, the solution was measured spectrophotometrically. The absorbance at 570 nm was measured with a microplate reader (Tecan, model Sunrise Remote, Männedorf, Switzerland) and background subtraction was adjusted with absorbance readings at 690 nm. The absorbance values were normalized by setting the negative control group (untreated cells) in each row to 100%. Subsequently, the viability of the treated cells was expressed as a percent of untreated cells.

### 2.6. Quality Check of Assay

According to the ISO 10993-5 standard, a test meets acceptance criteria if the left (column 2) and the right (column 11) mean of the blanks do not differ by more than 15% from the mean of all blanks; this criterion was met in all our experiments. Additionally, the standard deviation of the untreated and each treated sample should not exceed 18%. The highest standard deviation observed was 12.9% for the regular-voltage experiments and 10% for the high-voltage experiments. Finally, the absolute value of optical density, OD_570_, obtained in the untreated wells indicates whether the 1 × 10^4^ cells seeded per well have grown exponentially with normal doubling time during the two days of the assay. The OD_570_ of untreated cells were ≥0.2 in all experiments, meeting the acceptance criteria of ISO 10993-5.

### 2.7. Statistical Analysis

All data are reported as mean ± standard deviation. One-way analysis of variance (ANOVA) was used for comparison of percent viability between different extract concentrations of the same sample. If statistically significant differences were found, post-hoc analysis was performed with Bonferroni test to determine which extract concentrations had different effects on viability. Paired *t*-test was used to examine the difference in viability between low-voltage and high-voltage experiments. Independent sample *t*-test was used to assess whether nicotine concentration was associated with differences in viability, with EC samples divided into two categories: low-nicotine (6–11 mg/mL, 9 samples) and high nicotine (12–24 mg/mL, 11 samples); the analysis was performed only for the low-voltage samples, since the number of samples tested in the high-voltage experiments was not sufficient to show any significant differences. Inhibitory concentration 50 (IC_50_, the concentration of extract that produced 50% viability) was estimated from regression plots. No observed adverse effects level (NOAEL) was defined as the highest extract concentration that showed statistically insignificant difference in viability compared to the 6.25% extract concentration. According to UNI ISO 10993-5 standard definition, viability of less than 70% by MTT assay was considered cytotoxic. All analyses were performed with commercially available software (SPSS v18.0, Chicago, IL, USA) and a two-tailed *P* value of ≤0.05 was considered statistically significant.

## 3. Results

### 3.1. Cell Viability from Exposure to CS and EC Vapor Generated at Low Voltage

For the low-voltage experiments, myocardial cell viability measurements for each EC vapour and CS extracts at different dilutions are displayed in Table 2. From the 20 samples tested, four samples exhibited a cytotoxic effect in the 3.7 volts experiments: “Cinnamon-Cookies” flavour was slightly cytotoxic at the highest extract concentration, while both samples of “El Toro Cigarillos” and “El Toro Puros” were cytotoxic at both 100% and 50% extract concentration. The range of myocardial cell survival for all EC samples at 3.7 volts was: 89.7%–112.1% at 6.25%, 90.6%–115.3% at 12.5%, 81.0%–106.6% at 25%, 7.4%–106.8% at 50% and 2.2%–110.8% at 100% extract concentration. The “base” sample was not cytotoxic at any extract concentration. CS extract was significantly cytotoxic at concentrations above 6.25%, with viability rate being: 76.9 ± 2.0% at 6.25%, 38.2 ± 0.6% at 12.5%, 3.082 ± 0.2% at 25%, 5.2 ± 0.8% at 50% and 3.9 ± 0.2% at 100% extract concentration. 

**Table 2:** Myocardial cell viability in cigarette smoke extract and in electronic cigarette vapour extracts produced at 3.7 volts.

			Dilutions			
Samples-nicotine (mg/mL)	100% ^a^	50% ^b^	25% ^c^	12.5% ^d^	6.25% ^e^	*p* *
Base-0	105.1 ± 1.2	103.5 ± 1.9	101.3 ± 4.2	100.7 ± 3.4	100.4 ± 2.3	0.251
Golden Margy-6	89.2 ± 0.2	93.0 ± 2.2	92.1 ± 1.3	95.3 ± 3.6	93.0 ± 6.3	0.361
RY69-6	98.9 ± 4.6	101.2 ± 5.4	96.0 ± 13.0	100.5 ± 2.7	100.2 ± 9.2	0.932
City-6	93.6 ± 2.5	89.4 ± 4.2	94.6 ± 2.3	93.3 ± 2.3	93.8 ± 2.8	0.282
Cinnamon Cookies-6	64.8 ± 2.5	100.8 ± 2.0	97.2 ± 2.9	99.3 ± 1.7	99.2 ± 3.8	<0.001
Golden Virginia-8	86.6 ± 1.8	89.1 ± 1.0	94.2 ± 3.0	95.5 ± 0.7	97.1 ± 1.4	<0.001
RY4-9	73.8 ± 3.7	106.6 ± 1.1	104.4 ± 1.9	103.6 ± 4.0	100.7 ± 0.8	<0.001
MaxBlend-9	104.4 ± 1.6	102.4 ± 2.0	102.4 ± 2.8	101.2 ± 7.6	102.7 ± 2.0	0.901
Americano-9	85.0 ± 2.0	98.3 ± 1.7	90.9 ± 4.4	94.7 ± 3.5	94.1 ± 5.9	0.017
American Tobacco-11	109.0 ± 1.6	106.8 ± 0.5	104.9 ± 1.0	101.3 ± 3.1	103.6 ± 2.5	0.007
Tribeca-12	110.8 ± 2.8	103.9 ± 5.5	106.6 ± 7.9	102.4 ± 5.1	101.7 ± 3.0	0.268
Green apple-12	106.6 ± 2.0	106.8 ± 2.0	105.2 ± 3.3	103.6 ± 4.5	99.2 ± 2.5	0.060
El Toro Cigarrillos-12(1) ^f^	39.1 ± 1.2	52.5 ± 1.8	81.0 ± 2.0	92.6 ± 0.4	99.2 ± 1.0	<0.001
El Toro Cigarrillos-12(2) ^f^	22.3 ± 4.0	66.9 ± 6.2	104.1 ± 5.8	109.9 ± 6.0	112.0 ± 8.8	<0.001
Silverberry-12	108.2 ± 8.5	107.2 ± 2.7	106.0 ± 1.7	103.2 ± 0.7	100.3 ± 2.0	0.200
Virginia-18	82.1 ± 0.8	95.8 ± 8.6	95.1 ± 3.0	90.6 ± 7.0	93.3 ± 8.5	0.136
Classic-18	95.0 ± 5.1	104.0 ± 9.1	101.1 ± 12.9	107.3 ± 8.3	89.7 ± 6.4	0.176
Tobacco echo-18	96.1 ± 5.0	96.4 ± 7.7	101.7 ± 3.1	102.7 ± 4.7	96.3 ± 7.3	0.479
Bebeka-18	75.7 ± 8.6	87.5 ± 2.2	90.8 ± 1.6	95.9 ± 1.9	99.0 ± 2.3	<0.001
El Toro Guevara-18 ^f^	84.5 ± 3.0	91.0 ± 3.5	94.6 ± 1.3	98.8 ± 2.0	102.5 ± 1.7	<0.001
El Toro Puros-24 ^f^	2.2 ± 0.6	7.4 ± 3.9	84.5 ± 6.5	115.3 ± 11.7	111.9 ± 7.4	<0.001
CS ^g^	3.9 ± 0.2	5.2 ± 0.8	3.1 ± 0.2	38.2 ± 0.6	76.9 ± 2.0	<0.001

Values are presented as mean ± standard deviation. Viability is expressed as percent, compared to untreated cells; **^a–e^** For electronic cigarette liquid extracts, dilutions represent (w/v): a, 1%; b, 0.5%; c, 0.25%; d, 0.125%; e, 0.0625%; **^f^** Electronic cigarette samples made by using tobacco leaves; **^g^** CS = cigarette smoke; * For every sample, a separate ANOVA was performed to compare survival between different extract dilutions of the sample.

**Figure 2:**
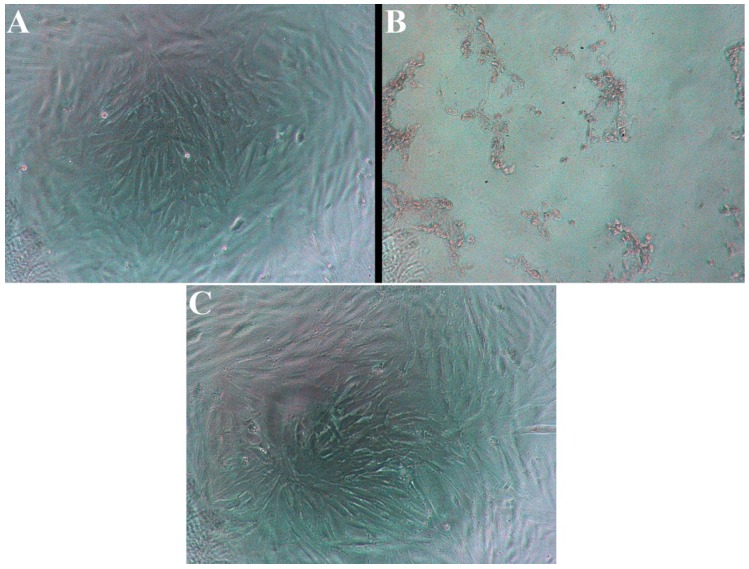
Microscopic images of cell cultures after 24 h treatment with: (**A**) untreated culture medium; (**B**) cigarette smoke extract at 100% extract concentration; (**C**) electronic cigarette vapour extract at 100% extract concentration (“American Tobacco”).

Examples of microscopic images of the cells after 24 h incubation in control medium, CS extract (100% concentration) and EC vapour extract (100% concentration) are displayed in Figure 2. Cells cultured in clear medium (Figure 2(A)) showed no sign of relevant mortality nor morphological alterations. Cells exposed to CS extract (Figure 2(B)) suffered from large scale cell death, as visible from the several apoptotic bodies and the absence of any surviving, morphologically stable cardiomyoblasts. Cells exposed to EC vapour extract-saturated medium (Figure 2(C)—American Tobacco) showed similar morphology to cells cultured in clear cell medium. Cell viability was determined to be approximately 100% for both clear medium culture and the EC vapour extract sample shown in the image.

### 3.2. Cell Viability from Exposure to EC Vapor Generated at High Voltage

For the high-voltage experiments, myocardial cell viability measurements for the EC vapour samples (n = 4) are displayed in Table 3. Range of viability at 4.5 volts was: 85.0%–97.7% at 6.25%, 83.5%–104.9% at 12.5%, 81.6%–105.7% at 25%, 83.4%–103.6% at 50% and 72.9%–95.4% at 100% extract concentration. The absolute mean difference in viability between 3.7 and 4.5 volts experiments was: 7.1 ± 4.1% at 6.25%, 5.0 ± 5.3% at 12.5%, 4.2 ± 4.8% at 25%, 5.0 ± 3.8% at 50% and 17.0 ± 12.2% at 100% extract concentration. Only the difference at 6.25% extract concentration was statistically significant (*p* = 0.039). None of the 4 samples was considered cytotoxic.

**Table 3:** Comparison of myocardial cell viability between regular and high voltage-produced electronic cigarette vapour extracts.

Samples-nicotine (mg/mL)	Voltage				Dilutions		
			100% ^a^	50%^ b^	25%^ c^	12.5%^ d^	6.25%^ e^
Golden Margy-6	3.7	89.2 ± 0.2		93.0 ± 2.2	92.1 ± 1.3	95.3 ± 3.6	93.0 ± 6.3
	4.5	82.2 ± 1.2		83.4 ± 3.6	81.6 ± 1.3	83.5 ± 2.4	85.0 ± 3.1
MaxBlend-9	3.7	104.4 ± 1.6		102.4 ± 2.0	102.4 ± 2.8	101.2 ± 7.6	102.7 ± 2.0
	4.5	95.4 ± 2.0		97.7 ± 1.8	100.6 ± 2.1	96.4 ± 2.5	97.7 ± 5.5
Tribeca-12	3.7	110.8 ± 2.8		103.9 ± 5.5	106.6 ± 7.9	102.4 ± 5.1	101.7 ± 3.0
	4.5	92.7 ± 2.7		103.6 ± 2.6	101.6 ± 3.0	97.7 ± 1.7	89.3 ± 4.7
Green apple-12	3.7	106.6 ± 2.0		106.8 ± 2.0	105.2 ± 3.3	103.6 ± 4.5	99.2 ± 2.5
	4.5	72.9 ± 3.5		101.3 ± 10.0	105.7 ± 3.3	104.9 ± 0.9	96.2 ± 0.7
*p* value *		0.069		0.080	0.175	0.156	0.039

Values are presented as mean ± standard deviation. Viability is expressed as percent, compared to untreated cells; **^a^**^–**e**^ Dilutions represent (w/v): a, 1%; b, 0.5%; c, 0.25%; d, 0.125% ; e, 0.0625%; * *p* value for comparison between different voltages at each dilution. For every extract dilution, separate paired *t*-tests were performed to compare cell survival between low and high voltage.

### 3.3. Nicotine Effects on Myocardial Cell Viability

The effects of nicotine concentration on cell survival are displayed in Table 4. No statistical significant difference in viability was observed according to nicotine concentration of the EC samples, indicating that nicotine content had no effect on myocardial cell survival.

**Table 4:** Myocardial cell viability according to nicotine concentration of the electronic cigarette samples tested at 3.7 volts (6.2 watts).

Viability according to nicotine concentration (mg/mL)			
Extract concentrations	6–11 (n = 9)	12–24 (n = 11)	*p* *
100%	89.5 ± 14.1%	74.8 ± 37.1%	0.247
50%	98.6 ± 6.7%	83.6 ± 30.6%	0.141
25%	97.4 ± 5.2%	97.3 ± 8.9%	0.981
12.5%	98.3 ± 3.7%	102.0 ± 7.3%	0.181
6.25%	98.1 ± 3.7%	100.5 ± 6.8%	0.357

Values are presented as mean ± standard deviation. Viability is expressed as percent, compared to untreated cells. * *p* value for comparison between different nicotine concentrations in each extract concentration. For every extract dilution, separate independent-sample *t*-tests were performed to compare cell survival between different nicotine concentrations groups at each extract dilution.

### 3.4. IC_50_ and NOAEL for EC and CS

IC_50_ and NOAEL for EC and CS samples are shown in Table 5. IC_50_ could be determined only for CS extract and for “El Toro Cigarrillos” and “El Toro Puros”, since for every other EC sample viability was higher than 50% at all extract concentrations. For 12 of the 20 samples at 3.7 volts, the “base” sample and two of the four samples at 4.5 volts, viability was not statistically different between 6.25% and any other extract concentrations; thus, NOAEL for these samples was defined as 100% extract concentration. The lowest NOAEL and IC_50_ were observed in CS extract.

**Table 5:** Inhibitory concentration 50 (IC_50_) and no adverse effect level (NOAEL) for each electronic cigarette extract and for cigarette smoke extract.

	Dilutions		
Samples-nicotine (mg/mL)	IC_50_	NOAEL
Base-0	>100%	100%
Golden Margy-6	>100%	100%
Golden Margy-6 *	>100%	100%
RY69-6	>100%	100%
City-6	>100%	100%
Cinnamon Cookies-6	>100%	50%
Golden Virginia-8	>100%	25%
RY4-9	>100%	50%
MaxBlend-9	>100%	100%
MaxBlend-9 *	>100%	100%
Americano-9	>100%	100%
American Tobacco-11	>100%	100%
Tribeca-12	>100%	100%
Tribeca-12 *	>100%	12.5%
Green apple-12	>100%	100%
Green apple-12 *	>100%	50%
El Toro Cigarrillos-12(1) ^a^	52%	6.25%
El Toro Cigarrillos-12(2) ^a^	69%	25%
Silverberry	>100%	100%
Virginia-18	>100%	100%
Classic-18	>100%	100%
Tobacco echo-18	>100%	100%
Bebeka-18	>100%	50%
El Toro Guevara-18 ^a^	>100%	12.5%
El Toro Puros-24 ^a^	36%	12.5%
CS ^b^	11%	6.25%

**^a^** Electronic cigarette samples made by using tobacco leaves; **^b^** CS = cigarette smoke; * Electronic cigarette vapour samples prepared at 4.5 volts.

## 4. Discussion

This is the first study that has evaluated the cytotoxic potential of EC vapour on cultured myocardial cells. High-voltage vaping, which is increasingly popular in EC users [20], was also tested for the first time. Importantly, a standardized protocol was used (ISO 10993-5), which defines cytotoxicity as viability <70% compared to untreated cells. EC samples were tested in vapour form which was produced by activating a commercially-available device, simulating the way ECs are used by every user. Finally, the same methodology was used to examine CS cytotoxicity. This is important since ECs are marketed for smokers only, as an alternative habit; therefore, the purpose of this study was to address the main scientific question which is whether ECs are less harmful compared to tobacco cigarettes. The main findings were that four out of 20 EC samples were cytotoxic on cultured myocardial cells, with most (but not all) tobacco-produced samples showing the lowest cell survival rate. Although samples of vapour produced with high voltage were not cytotoxic, cell viability was reduced compared to vapour produced with regular voltage. Overall, CS was significantly more cytotoxic, with toxicity observed even when CS extract was diluted to 12.5% of original concentration.

Several studies have shown that CS extract has direct necrotic and apoptotic effects on cardiac myocytes [10,21]. The main mechanism responsible is oxidative stress [22] and inflammation [23]. Mitochondrial dysfunction and DNA damage also play an important role in causing cell damage [24,25,26]. CS is a complex suspension containing more than 4,000 chemicals [27]. Several of them have been studied separately and were found responsible for cytotoxic effects, such as acrolein [28], acetaldehyde [29], formaldehyde [30], and heavy metals [31]. The results of this study are in line with previous observations about the cytotoxic effects of CS extract.

Propylene glycol and glycerol are the main ingredients of EC liquids. Both are classified by Food and Drug Administration (FDA) and by the Flavor and Extracts Manufacturers Association (FEMA) as additives that are “generally recognized as safe” for use in food (FEMA GRAS numbers 2,940 and 2,525 respectively). They are also used in tobacco cigarettes as humectants; however they may be pyrolyzed to acrolein and formaldehyde [32,33]. Goniewicz *et al.* found acrolein and formaldehyde in EC vapour [34]; however, the levels detected were lower compared to CS by orders of magnitude, probably because the temperature of evaporation of EC liquid is lower compared to the temperature of combustion in tobacco cigarettes. Similar observations were made by Lauterbach and Laugesen [35]. Even if such chemicals were released during vapour production in this study, the amount was probably not enough to produce any significant cytotoxic effect on cultured cells. Nicotine, at levels commonly found in cigarettes, does not induce cell death and may even have anti-apoptotic properties in myocardial [36] and other cell lines [37,38]. In this study, cell viability was independent of the nicotine concentration in EC liquid samples.

Out of 20 EC samples tested at regular voltage, four were found to be cytotoxic on the tested cell line. Cell survival after exposure to “Cinnamon and Cookies” EC extract was just below the level defined as cytotoxic. Studies have shown that cinnamon oils may have anti-oxidative and anti-inflammatory properties [39]. However, there are several reports in internet EC user forums that cinnamon flavours may have some irritant effects when inhaled; this may be due to an allergic reaction [40] rather than a cytotoxic effect. No studies have evaluated the effect of cinnamon extracts on cardiomyocyte survival. Cinnamaldehyde, the main ingredient of cinnamon flavouring, is heat unstable. Benzaldehyde can be produced if heated above 60 °C [41], which may have cytotoxic properties on cultured cells [42]. Cells survival was significantly reduced from exposure to “El Toro Cigarrillos” and “El Toro Puros” EC extracts. It is reasonable to assume that the cytotoxicity observed may be due to the use of cured tobacco leaves in the production process. The possibility that several tobacco impurities may be present in the final sample, despite being filtered before bottled, cannot be excluded. The two samples of “El Toro Cigarrillos” liquid tested were equally cytotoxic, therefore excluding the possibility of experimental error or material dysfunction. However, no cytotoxicity was observed in another sample (“El Toro Guevara”) produced by the same company with similar methodology. According to the company’s website (www.houseofliquid.com), different blends of tobacco leaves are used in these liquids. The difference in cell viability could be attributed to differences in tobacco blends, possibly related to the curing process or pesticides used during cultivation of tobacco [43]. Although other manufacturers reported the use of industrially-produced tobacco extracts in several of the samples tested, none of them was found to be cytotoxic on cultured cells. Further studies need to be conducted in order to determine qualitative or quantitative differences in chemical composition of the vapour of these EC liquids that could be associated with differences in cell survival; no such analysis was performed in this study.

EC devices using higher voltage and wattage for vapour production have been developed in recent years. Consumers report perceiving additional pleasure from high-voltage EC use [20], probably due to more vapour production and different flavour of the resulting vapour. Results from the limited number of samples tested showed that cell viability was reduced; the difference was not statistically significant for most extract concentrations, but this should probably be attributed to the low number of samples tested. It is expected that higher energy applied to the resistance of the EC device will result in higher evaporation rate of the liquid; this can result in temperature elevation, especially if the liquid supply to the wick and resistance is not sufficient [12]. Vapour was produced by lower puff duration compared to regular-voltage experiments because that was the duration that could be used in realistic settings without reproducing the dry-puff phenomenon. Further studies are needed to clarify the cytotoxic potential of high-voltage EC use, by examining more samples and using more efficient atomisers.

The results of this study raise the possibility that cytotoxicity depends on flavourings rather than other ingredients of EC liquid. A previous study by our group showed that one of 21 liquids had cytotoxic properties on cultured fibroblasts [11]. In that study, all samples were produced by the same manufacturer and had the same main ingredients (propylene glycol, glycerol and nicotine in similar concentrations). Therefore, the difference in cell viability could only be attributed to the flavouring. Although some of the flavourings are approved for use in foods, their effects are unknown when heated and evaporated. Moreover, manufacturers use different quantities of flavourings in the EC liquids, thus it cannot be excluded that cytotoxicity may depend on the quantity rather than on the flavouring itself. Unfortunately, there is no way to predict which flavourings may have a cytotoxic effect unless they are specifically examined. Considering the huge variety of liquids currently available in the market, it may be essential to test all flavourings and determine the flavouring concentrations that can be considered safe. A study by Bahl *et al.* also found an association between EC liquid cytotoxicity and flavourings [44]. However, they evaluated the samples in liquid form. Such tests should preferentially be done in vapour form, produced by activation of an EC device, since this represents the pragmatic way these liquids are used by consumers. Despite the findings of cytotoxicity in some of the flavours, eliminating all flavourings from ECs would be controversial, since they seem to play an important role in ECs’ acceptance [20]; liquids containing just glycerol, propylene glycol and nicotine would be virtually flavourless, making them less appealing to smokers as a smoking substitute.

Some limitations apply to this study. Cytotoxicity studies on cultured cells have been developed in order to reduce the use of experimental animals. Extrapolating these results to the human *in vivo* toxicity should be done with caution; clinical studies are necessary in order to confirm findings from cytotoxic studies. However, a comparative measure of toxicity with CS extract was provided, which has well-established *in vivo* toxic effects. There is no consensus on the methodology of preparing and testing EC vapour extracts; therefore, a standardised method was used, which specifically defines the cell survival rate that is considered cytotoxic. Nicotine levels in the extracts were not measured. However, the purpose of the study was to evaluate the effects of EC vapour by simulating realistic conditions of use rather than by setting a pre-specified level of nicotine content in the extract. Therefore, commercially available EC batteries and atomiser were used to produce vapour, and they were previously evaluated by an experienced user in order to ensure that the experimental conditions would not represent unrealistic situations (such as the dry puff phenomenon). Although there is no established comparative measure between EC and tobacco cigarette use and no method to standardise the vapour extracts of EC liquids in a similar way to CS extracts, the decision to compare extract from three tobacco cigarettes with 200 mg of liquid was based on previous observations from our group indicating that 5 min of EC use by experienced consumers (which is the time needed to smoke one tobacco cigarette) leads to consumption of approximately 60 mg of liquid [12]. Additionally, it should be emphasized that the results are not applicable to every EC liquid available to the market. It is possible that cell survival may be dependent on nicotine concentration if low-quality nicotine is used for EC liquid production, which would contain significant amounts of tobacco impurities. The same applies for other liquid constituents [45]. Finally, studies on the underlying causes for the difference in cytotoxic potential of EC samples should be undertaken, evaluating the quality and quantity of flavourings used among other factors. This study examined only the end-result of exposure, without evaluating the cause for the differences in cell survival.

## 5. Conclusions

In conclusion, from 20 commercially-available EC liquids that were tested in vapour form, four were found to be cytotoxic on cultured cardiomyoblasts. Cytotoxicity was mainly observed in most (but not all) samples produced by using tobacco leaves, while one sample using food-approved flavouring was marginally cytotoxic. EC vapour production by using higher-voltage devices caused a decrease in cell survival. Overall, EC vapour extracts showed significantly higher cell viability compared to CS extract, based on a realistic-use rather than a standardized comparative level of exposure. This supports the concept that ECs may be useful as tobacco harm reduction products; however, more studies are needed, especially in clinical level, in order to evaluate the effects of EC use on human health.
